# Human parvovirus 4 ‘PARV4’ remains elusive despite a decade of study

**DOI:** 10.12688/f1000research.9828.1

**Published:** 2017-01-27

**Authors:** Philippa C. Matthews, Colin Sharp, Peter Simmonds, Paul Klenerman

**Affiliations:** 1 Nuffield Department of Medicine, University of Oxford, Peter Medawar Building for Pathogen Research, South Parks Road, Oxford, OX1 3SY, UK; 2 Department of Infectious Diseases and Microbiology, Oxford University Hospitals NHS Foundation Trust, John Radcliffe Hospital, Headley Way, Headington, Oxford, OX3 9DU, UK; 3 Roslin Institute, University of Edinburgh, Easter Bush, Midlothian, EH25 9RG, UK; 4 NIHR Biomedical Research Centre, John Radcliffe Hospital, Headley Way, Headington, Oxford, OX3 9DU, UK

**Keywords:** Human parvovirus 4, PARV4, PARV4 phylogeny, PARV4 biology, tetraparvovirus

## Abstract

Human parvovirus 4 (‘PARV4’) is a small DNA tetraparvovirus, first reported in 2005. In some populations, PARV4 infection is uncommon, and evidence of exposure is found only in individuals with risk factors for parenteral infection who are infected with other blood-borne viruses. In other settings, seroprevalence studies suggest an endemic, age-associated transmission pattern, independent of any specific risk factors. The clinical impact of PARV4 infection remains uncertain, but reported disease associations include an influenza-like syndrome, encephalitis, acceleration of HIV disease, and foetal hydrops. In this review, we set out to report progress updates from the recent literature, focusing on the investigation of cohorts in different geographical settings, now including insights from Asia, the Middle East, and South America, and discussing whether attributes of viral or host populations underpin the striking differences in epidemiology. We review progress in understanding viral phylogeny and biology, approaches to diagnostics, and insights that might be gained from studies of closely related animal pathogens. Crucial questions about pathogenicity remain unanswered, but we highlight new evidence supporting a possible link between PARV4 and an encephalitis syndrome. The unequivocal evidence that PARV4 is endemic in certain populations should drive ongoing research efforts to understand risk factors and routes of transmission and to gain new insights into the impact of this virus on human health.

## Introduction

Human parvovirus 4 (‘PARV4’) was first described in 2005 ^1^, but many important features of its epidemiology, transmission, and clinical significance remain elusive ^2^. PARV4 is a small non-enveloped, single-stranded DNA virus with a genome of approximately 5 kb, translated as two major and two minor open reading frames. Classified as a member of the genus *Tetraparvovirus*, in the family *Parvoviridae* (sub-family *Parvovirinae*), it sits phylogenetically alongside porcine and bovine hokoviruses ^3^, while its closest human-tropic relatives are well-characterised B19V (genus *Erythroparvovirus*) and human bocaviruses (genus *Bocavirus*) ( Table 1).

**Table 1.** Potential and confirmed disease associations of parvoviruses in human and animal hosts.

Parvovirus B19	Bocaviruses	Bovine and porcine hokoviruses	Human parvovirus 4 (‘PARV4’)
• Childhood rash and fever • Inflammatory and autoimmune sequelae, including myocarditis, arthritis, and glomerulonephritis • Myelosuppression leading to anaemia and intra-uterine hydrops • Rarely encephalitis	• Respiratory infections, predominantly in children ^58^	• Wasting syndromes ^52^ • Foetal infection/abortion • Diarrhoea • Skin disease • Arthritis	• Influenza-like symptoms ^1^ • Encephalitis ^32, 39^ • Transient rash and hepatitis ^11^ • Foetal hydrops ^24^ • Transmission via respiratory secretions or faeces suggestive of respiratory tract infection or gastroenteritis ^15^ • Acceleration of progression to AIDS in HIV-infected adults ^8^

The evidence of clinical associations for B19V (left-most column) are robust and consistent, in contrast to those for animal hokoviruses and PARV4, in which the evidence is often at the level of case reports or small case series only and for which replication in other cohorts is lacking. Other parvoviruses (for example, dependoparvoviruses and protoparvoviruses) have also been detected from human samples, but evidence is lacking for pathogenicity ^59^.

Ongoing investigation of PARV4 is being driven mainly by several intriguing features of its distribution: it is endemic in certain geographic areas ^4^, but elsewhere is found confined only to certain high-risk groups ^5^. The first reported identification of PARV4 was from an injecting drug user with hepatitis B virus (HBV) infection, who was screened for viral infections following presentation with influenza-like symptoms ^1^. Based on this index case, subsequent studies in North America and Western Europe focused mainly on groups with risk factors for parenteral infection and those infected with other blood-borne viruses (BBVs). In these settings, a clear picture has emerged in which PARV4 infections are consistently and strongly associated with HIV, HBV and HCV, mostly in the setting of persons who inject drugs (PWIDs) and those with a history of multiple transfusion ^5– 10^. Despite attempts to identify it outside this setting, infection has not been detected in household contacts of seropositive individuals ^11^ and is infrequent in the general population ^6, 12^.

However, groups working in Africa have reported a completely different population epidemiology: in this setting, the detection frequency of PARV4 IgG (indicative of current or past exposure) ranges from 30% to 50% in the general population, without any consistent relationship with BBVs ^4, 13– 15^. Acute infections associated with viraemia were detected at a frequency of 8.5% among young children in Ghana ^16^.

**Figure 1.**
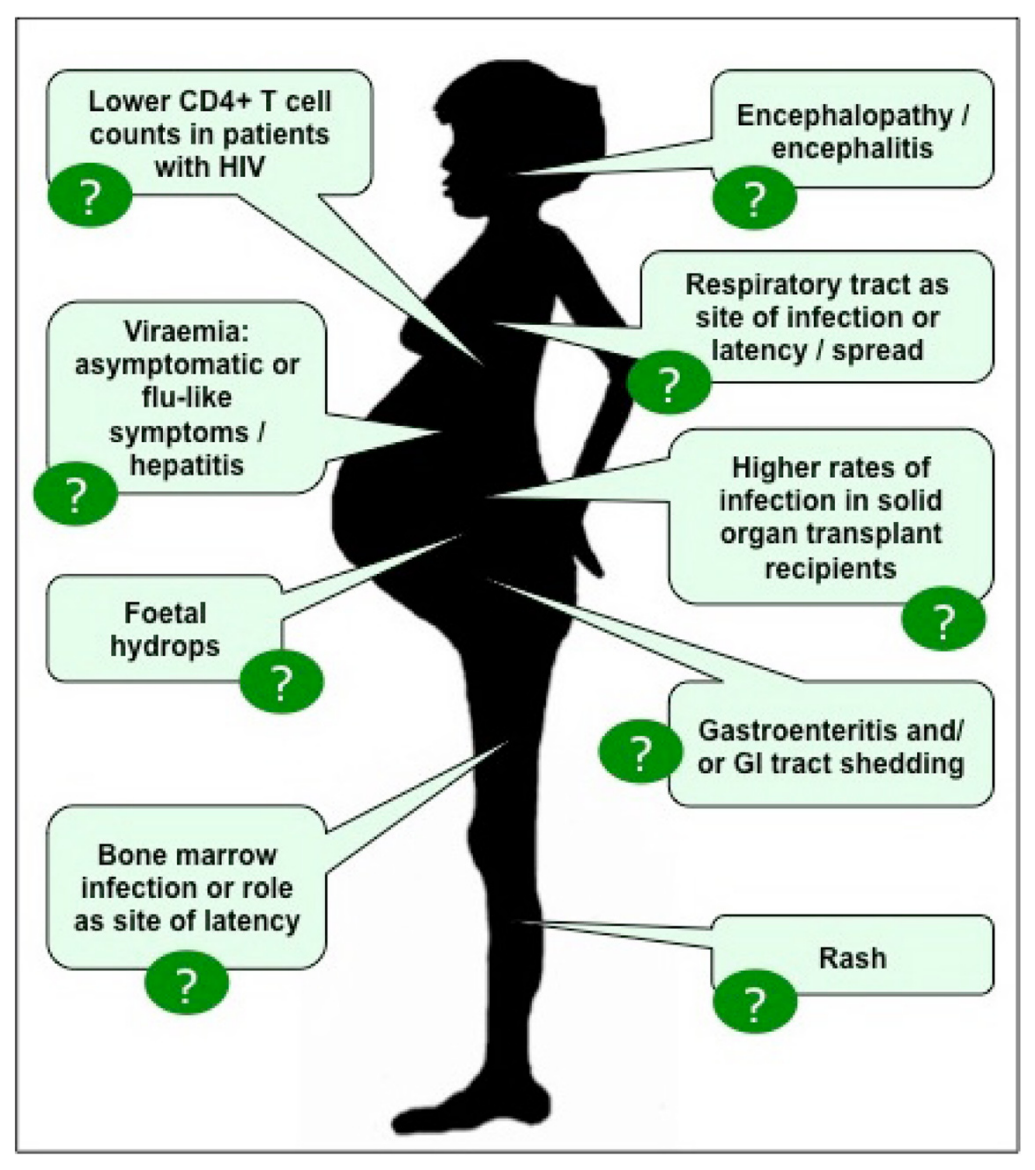
Cartoon depicting potential clinical associations of human parvovirus 4 (PARV4). The references for each of these are provided in Table 1. The question marks highlight that robust and consistent evidence is still lacking for all of these associations. GI, gastrointestinal; HIV, human immunodeficiency virus.

The clinical syndromes that have been described in association with PARV4 are summarised in Table 1 and Figure 1. However, there are too few data to make any of these associations secure, and attributing causality in a robust way is inevitably challenging.

This article seeks to summarise recent advances that have been made in understanding and characterising the epidemiology, immunology, phylogeny, and pathophysiology of PARV4. We have maintained a primary focus on updates made since our previous review of the topic in 2014 ^2^, although we have also drawn upon some older references where they are fundamental in supporting our current understanding. Crucially, there are still many gaps in the story; therefore, we have also set out to identify and highlight important questions for future study.

## New advances in the PARV4 literature

### Population prevalence of infection in different geographic locations

The most substantial updates to the PARV4 literature are studies to determine the prevalence of infection in geographic areas that had not previously been represented. This includes new studies in Scandinavia ^17, 18^, the Middle East ^19^, and South America ^20, 21^; we have summarised these data in Table 2. Below, we discuss these findings with further reference to their applicability to understanding risk factors for infection.

**Table 2.** Summary of human parvovirus 4 (PARV4) prevalence studies published 2014–2016.

Location of cohort	Characteristics of cohort	Prevalence of PARV4	PARV4 genotype	Clinical associations	Study
**Europe/Scandinavia**					
France	High-risk blood donors and recipients of blood products (including HCV-positive n = 216 and HIV-positive n = 314)	PARV4 IgG detected in 20% of HCV-positive, 23% of HIV-positive	Not determined	Not reported	Servant-Delmas *et al.* ^12^ (2014)
Denmark	HIV-positive children and adolescents (n = 46); 52% of African origin.	PARV4 IgG detected in 9%; IgM in 2%. PARV4 PCR-negative in all cases.	PCR done but negative in all cases	Lower CD4 ^+^ T-cell counts in PARV4 IgG- positive subjects	Rosenfeldt *et al.* ^17^ (2015)
Denmark	Birth cohort of healthy infants at birth (n = 176) and at 1 year (n = 202) and mothers (n = 228)	PARV4 IgG detected in 0.9% of mothers and in 0% of children at birth and at age 1 year	Not determined	Not reported	von Linstow *et al.* ^18^ (2015)
**Middle East**					
Iran	HIV-positive (n = 133) versus healthy blood donors (n = 120)	PARV4 DNA detected in 35% of HIV-positive and 17% of HIV-negative subjects	Sequences all consistent with genotype-1	Not reported	Asiyabi *et al.* ^19^ (2016)
**Africa**					
South Africa	HIV-positive mothers (n = 43), HIV-positive children (n = 90) and their HIV-negative siblings (n = 24)	PARV4 IgG detected in 37%. Trend towards higher prevalence in adults compared with children.	Not determined	No association between PARV4 status and CD4 ^+^ T count or HIV viral load	Matthews *et al.* ^23^ (2015)
**Asia**					
Taiwan	Healthy health-care workers (n = 10)	PARV4 IgG detected in 60% and IgM in 30% (persistent for 1-year follow-up period)	PCR products identical to a previous sequence ^31^, described as a ‘sub-cluster’ of genotype-2	Subjects were well or asymptomatic.	Chen *et al.* ^33^ (2015)
India	Patients with acute encephalitis syndrome (n = 10) after exclusion of other causes of viral encephalitis	PARV4 DNA detected in 20% in cerebrospinal fluid	Sequences consistent with genotype-2	Encephalitis	Prakash *et al.* ^39^ (2015)
**South America**					
Brazil	Patients with haemophilia (n = 28), beta-thalassaemia major (n = 40), and volunteer blood donors (n = 68)	PARV4 DNA detected in 6%	Genotype of sequences not reported	Not reported	Slavov *et al.* ^20^ (2015)
Brazil	HTLV1/2-positive patients (n = 130) ± HIV, HBV, HCV co-infection	PARV4 DNA detected in 5% (n = 1 with HCV and 1 with HCV/HIV)	Similar to Indian strain (GenBank HQ593532 ^32^) – genotype-2	Not reported	Slavov *et al.* ^21^ (2016)

HBV, hepatitis B virus; HCV, hepatitis C virus; HIV, human immunodeficiency virus; HTLV, human T-cell lymphotrophic virus; PCR, polymerase chain reaction.

### Risk factors for PARV4 infection

Based on the strong relationship between PARV4 and HIV, HBV, and HCV co-infection in North American and European populations, several studies have compared PARV4 exposure (screening for PARV4 IgG or DNA or both) in HIV-positive and HIV-negative groups in different settings. In Iran, HIV infection (with or without HCV co-infection) was reported to be associated with a high prevalence of PARV4 viraemia (35%) ^19^. However, in this study, the background prevalence of viraemia in healthy blood donors was also high at 17% ^19^. Higher seroprevalence was also reported in Scandinavian subjects in the setting of HIV infection; the PARV4 IgG detection frequency was 8% among HIV-positive children ^17^, while the background population seropositivity rate was less than 1% in adults and absent in children ^18, 22^. Stratification of risk factors in a French study reported the highest PARV4 prevalence in PWIDs, especially among those who were HIV-infected, but also among men who have sex with men (MSM), particularly those likely to have been exposed in the 1980s ^12^.

Our own study of a cohort of mothers and children in Kimberley, South Africa, was comparable to previous reports from sub-Saharan Africa in finding an overall PARV4 seroprevalence of 37% with no evidence that HIV infection was a risk factor ^23^. Although vertical transmission has previously been demonstrated in another setting ^24^, there was no concordance between maternal and child serostatus, suggesting that maternal transmission is probably not a major contributor to the overall burden of infection in South Africa ^23^. The clear trend towards increasing acquisition with age is consistent with a broader environmental source of PARV4 infection.

A potential association between PARV4 exposure and infection with another BBV, human T-cell lymphotrophic virus (HTLV), was described in a Brazilian study ^21^. Among 113 subjects with HTLV-1 infection, seven (6%) were positive for PARV4 DNA, two of whom also had HIV or HCV infection or both. Based on a history of blood transfusion in one of these individuals, it was postulated that this represents the most likely route of acquisition of all three viruses. However, the remaining five cases had no other co-infecting BBVs and no history of injecting drug use or blood transfusion, leading to the conclusion that other transmission routes of PARV4 transmission may also be operating ^21^. Another study published by the same group identified PARV4 viraemia in 2–5% of individuals either donating blood or receiving transfusions of blood products ^20^. Neither of these Brazilian studies screened for PARV4 IgG, so the prevalence of PARV4 exposure in this setting remains unknown ^20, 21^.

### Viral biology/immunology

**Figure 2.**
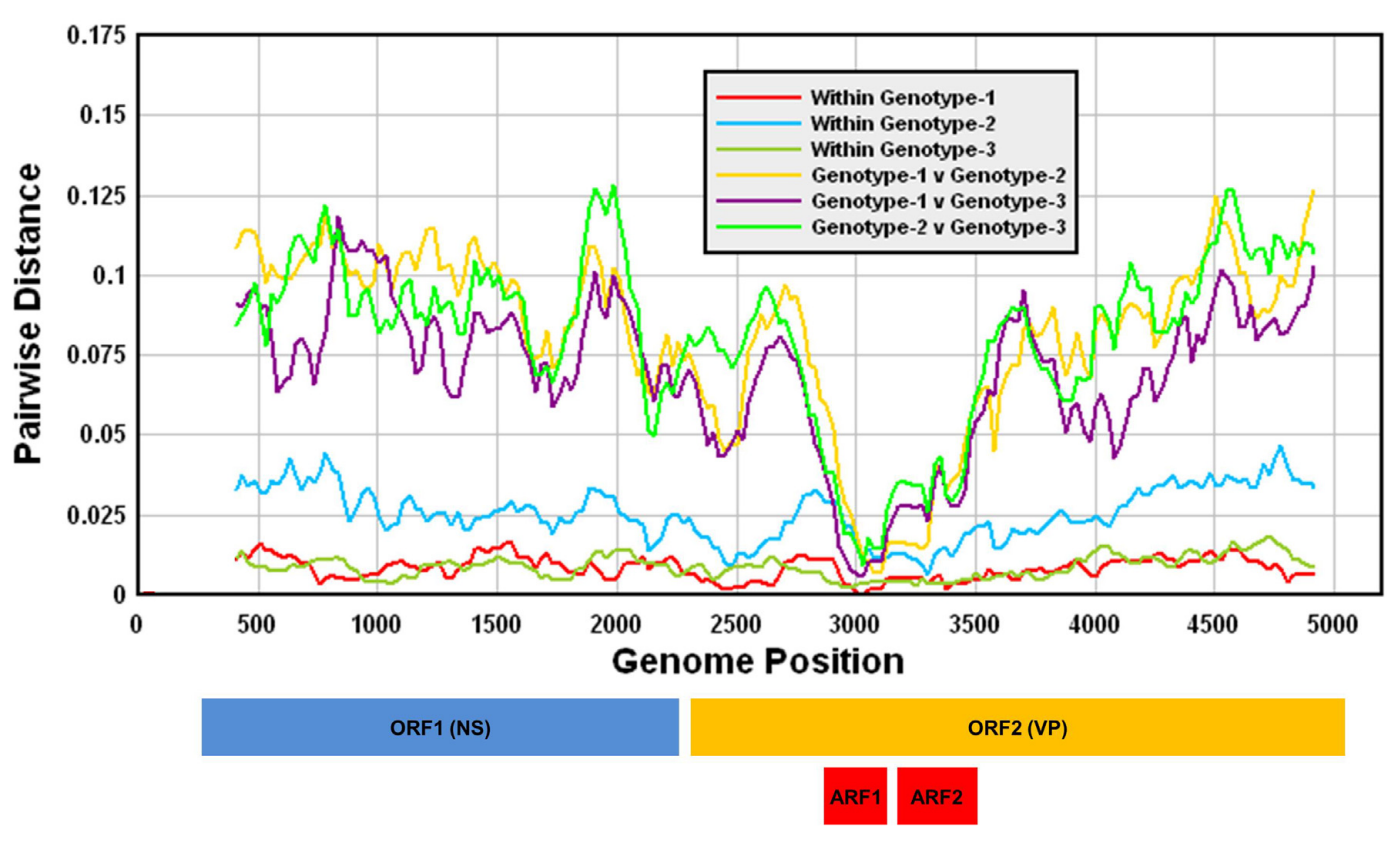
Variability scan of human parvovirus 4 (PARV4) genomes, showing mean pairwise nucleotide distances of sequential 250 base fragments, incrementing by 25 bases between data points. Sequence comparisons were between 10 genotype-1 (DQ873386, DQ873387, DQ873388, DQ873389, EU175856, EU200667, EU546204, EU546210, EU546211, and NC007018), 13 genotype-2 (DQ873390, DQ873391, EU175855, EU546205, EU546206, EU546207, HQ593530, HQ593532, KJ541119, KJ541120, KJ541121, KM390024, and KM390025), and seven genotype-3 (EU874248, JN798193, JN798194, JN798195, JN798196, KU871314, and KU871315) complete or near complete genome sequences. A genome diagram drawn to scale is included showing the main non-structural (NS, ORF1) and structural (VP, ORF2) gene coding regions as well as the positions of the additional reading frames (ARF1 and ARF2) embedded in ORF2. All nucleotide positions are numbered based on the reference sequence NC007018. It is striking that the region containing the two small ORFs is the most conserved of the whole genome; this may be in part a general feature of ORFs (where less flexibility is likely to be tolerated) but could also point to an important structural or functional role of this region. ORF, open reading frame.

**Figure 3.**
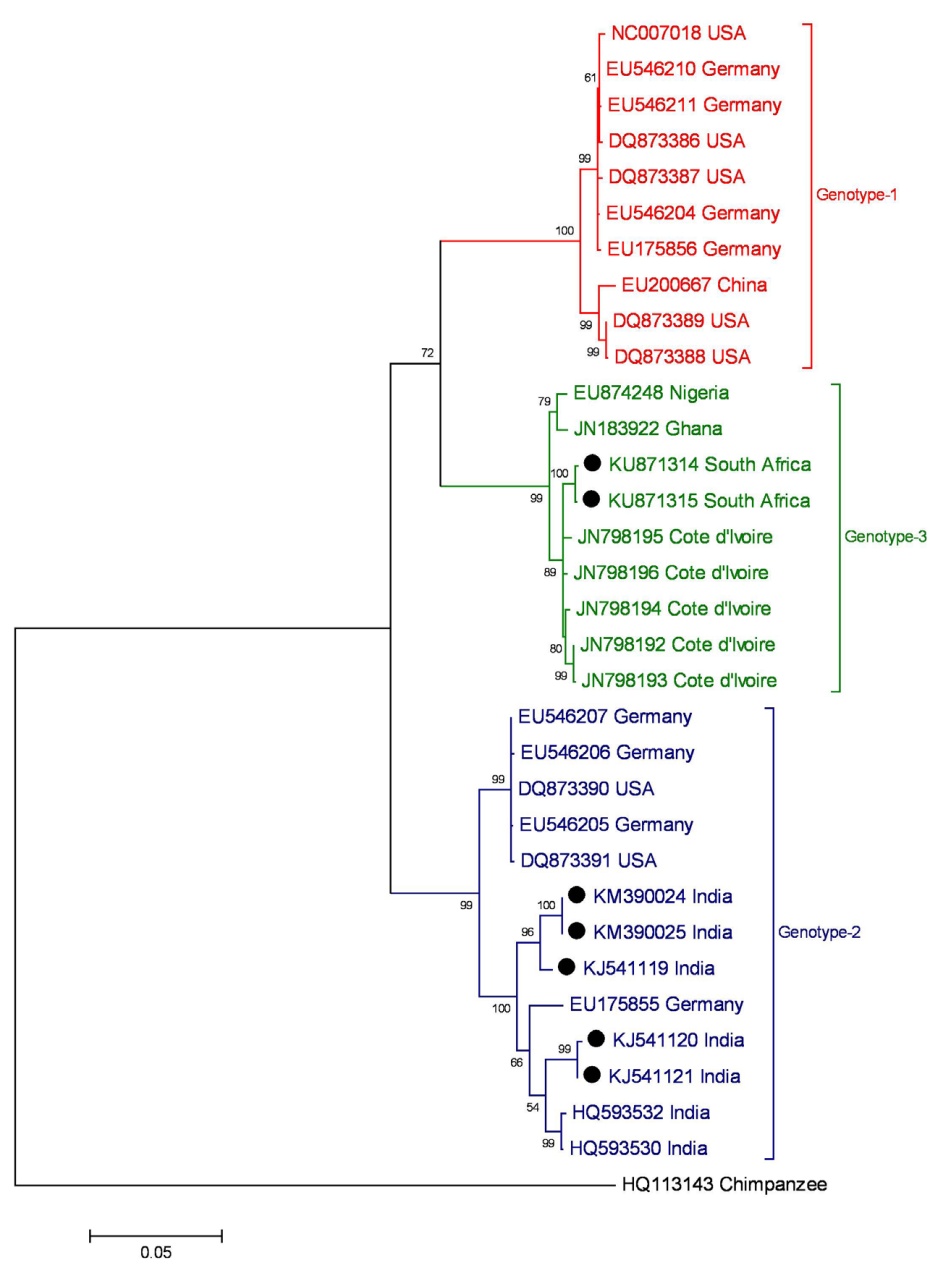
Phylogeny of published complete or near complete human parvovirus 4 (PARV4) genome sequences as inferred from complete NS (ORF1) nucleotide sequences (equivalent to nucleotides 283–2271 of the reference sequence NC007018). Sequences published since 2014 are highlighted with filled circles. The evolutionary history was inferred by using the maximum likelihood model. The optimum maximum likelihood model (lowest Bayesian information criterion score and typically greatest maximum likelihood value for the nucleotide sequence alignment) was first determined and used for phylogenetic reconstruction. This was the Tamura 3-parameter model with a gamma distribution. Bootstrap support of branches (500 replications) is indicated.

PARV4 variants characterised to date fall into three distinct genetic groups (genotypes 1–3) but show relatively restricted sequence variability; in both the non-structural (NS) and capsid-encoding genome regions, there is less than 3% amino acid (8–9% nucleotide) sequence divergence between genotypes (representations of diversity can be found in Figure 2 and in Supplementary data 1). The even greater restriction of sequence diversity within genotypes supports the hypothesis for relatively recent transmission to human populations ^5^. Relatively few new PARV4 sequences have been reported over the period that we have reviewed, and although incremental additions to the sequence database have been made over recent years ( Figure 3), these have not substantially extended the known diversity of this virus.

The distribution of the three genotypes is summarised as follows:

• Genotype-1 is the genotype currently predominant in Europe and North America ^11, 25– 27^. Sequencing of PARV4-positive plasma pools and blood products concentrates from various collection years ^28, 29^ as well as analysis of the age distributions of PARV4-infected study subjects ^26^ suggests that genotype-1 replaced genotype-2 as the predominantly acquired genotype in these regions from the mid-1990s onwards, analogous to the genotype replacement that has been described for another parvovirus, B19V ^30^. PARV4 sequences derived from a recent study in Iran also all grouped as genotype-1 ^19^.
• Genotype-2 (formerly known as PARV5) has been identified in European and North American cohorts ^25, 27^ and appears to be the more common genotype in individuals likely infected in the 1980s or early 1990s ^11, 26^. It was also the predominant strain reported from Asia ^31– 33^. Limited amplification of PARV4 genomes from Brazil ^21^ yielded sequences that were most similar overall to genotype-2 strains previously identified in India ^32^.
• Genotype-3 has been consistently identified from African cohorts ^13^. We have recently derived full-length PARV4 sequences from an adult and child in South Africa (GenBank accession numbers KU871314 and KU871315), which cluster together with genotype-3 sequences.

Various reports provide preliminary evidence that PARV4 may not be completely cleared after acute infection, suggesting that it has the potential for latency and reactivation. Two studies in Taiwan have sought to describe serological responses to PARV4 infection by undertaking longitudinal follow-up of their cohorts ^31, 33^. Among a small cohort of health-care workers, IgG seropositivity was strikingly high at 60%; half of these had a positive IgM that was sustained over the study period, and viraemia was detected at one of several sampling time-points ^33^. In the second study, this time focusing on a high-risk PWID cohort, IgM was reported as remaining positive for up to 21 months ^31^. Meanwhile, *in vitro* experiments have demonstrated a sustained high-magnitude *ex vivo* CD8 ^+^ T-cell response to PARV4 NS peptide ^34^. Prolonged IgM-positivity and sustained high-magnitude T-cell responses together suggest a sustained or relapsing/remitting exposure to viral antigen. Tissue homing and sites of latency are unknown, but existing data suggest that bone marrow, the respiratory tract, liver and gut might represent potential sites of viral replication ^5, 15, 26, 27, 35, 36^. Skin has also been highlighted as a potential reservoir site for B19V ^37^, although—to the best of our knowledge—no study has investigated this as a site of PARV4 replication. The role of capsid proteins in parvovirus infections has been recently reviewed ^38^, although there are no specific functional or structural data for PARV4.

### Clinical associations of PARV4

To date, determining the clinical impact (if any) of PARV4 infection remains perhaps the most uncertain area of research into this virus, despite the medical importance of establishing such disease associations. A wide range of potential infection outcomes has been proposed (summarised in Table 1), but the evidence for a pathogenic role of PARV4 in any of these has not been significantly expanded in recent years.

The most significant addition to the literature builds on an earlier report linking PARV4 with an encephalitis syndrome in Bellary, South India ^32^. Further efforts have been made to substantiate this important potential disease association by screening ten patients in Northern India with an acute encephalitis syndrome in whom other viral causes had been ruled out. The investigators identified PARV4 DNA in the cerebrospinal fluid (CSF) and blood of two ^39^. However, it remains uncertain whether PARV was actually the agent of the observed disease, or a bystander that was coincidentally detected in a highly exposed population. Neither this nor the previous study ^32^ included a control group in which the background incidence of PARV4 infections could have been evaluated. It is additionally possible that detection of PARV4 DNA in CSF in these two studies simply represents reactivation of the virus in the central nervous system in the context of other pathology.

### Developing and refining methods for the study of PARV4

One approach to the determination of PARV4 serostatus is to use an enzyme-linked immunosorbent assay (ELISA)-based approach with an optical density read-out that is calibrated as positive or negative. However, the choice of a fixed cut-off above which threshold samples are termed ‘positive’ can be problematic and evidently has bearing on the final estimation of prevalence; the choice of this threshold evidently affects sensitivity and specificity of the test, and may need to be altered in light of the consideration of the primary reason for screening (diagnosis versus seroepidemiology). This effect is exemplified by recently published simulations, alongside discussion of alternative approaches that set out to quantify population prevalence ^40^. One of these is to use ‘mixture models’, which estimate population seroprevalence from the complete screened dataset rather than classifying each individual sample as positive or negative ^40^. Application of these new analytical methods to existing PARV4 serosurvey datasets has yet to be performed but may refine prevalence estimates and allow for better comparison between studies.

A novel polymerase chain reaction (PCR) assay for genotyping and quantifying PARV4 was published in 2014 ^41^. The authors report that their method reliably discriminates between PARV4 genotypes, and use quantitative PCR to measure viraemia ^41^.

### Practical implications: human tissue for transfusion and transplantation

Consistently high detection frequencies of anti-PARV4 antibodies in sera of haemophiliac individuals provide strong evidence for some transmission via the use of clotting factor concentrates ^11, 42^. Although modern procedures for viral inactivation may effectively remove parvovirus infectivity, PARV4 is more robust than B19V to heat and other virus inactivation methods used in blood product manufacture, and there is ongoing discussion about the need to implement donation screening for PARV4 DNA and/or to enhance measures to ensure that the virus is eradicated from blood products for transfusion ^10, 43– 45^.

Blood donors have been screened as a component of several studies (see representative data in Table 1 for recent examples). PARV4 IgG screening of UK blood donors identified a seropositivity rate of 4.8%, but no donations were PCR-positive ^10^. However, the results of such studies are disparate, as a previous study from the same group did find PARV4-positive pools ^46^. Studies outside the UK that have screened blood donors by PCR have found frequencies of 0% in Italy ^35^; 3–4% in Brazil ^20^, Thailand ^9^, and North America ^25^; and as high as 17% in Iran ^19^.

Solid organ transplant recipients are a more difficult population to assess and are unsurprisingly less well represented in the literature; no new articles on this topic have been published in the past two years. However, since 2008, an Italian study has reported finding PARV4 in a small number of renal transplant recipients ^35^, and a French cohort of lung transplant patients reports that 14% were viraemic ^47^. The clinical significance of these findings and whether these cases represent reactivation of autologous virus in the context of immunosuppression or transmission of donor virus at the time of organ transplantation have yet to be explored.

### Insights from new animal data

There is increasing evidence that infections with porcine and bovine hokoviruses are endemic in both wild and domestic animals; most of these recent animal data are simply detection studies and do not provide any new data about pathogenicity. These include the frequent detection of porcine hokovirus in hunted wild boar in Portugal ^48^ and a bovine hokovirus in domestic yaks ^49^. In South America, another hokovirus was found to be endemic in a survey of Brazilian pig herds, in which post-weaning multi-systemic wasting syndrome was common ^50^, and a novel porcine hokovirus (referred to as porcine parvovirus 6, or PPV6) has been associated with foetal loss in pigs in China ^51, 52^.

As livestock pathogens, these organisms are potentially implicated as a cause of significant economic losses in agriculture ^52, 53^. However, since most of the suggested pathogenicity has been attributed to protoparvoviruses or copiparvoviruses, we cannot necessarily infer any significance to tetraparvoviruses. Nevertheless, the widespread isolation of parvoviruses, particularly from domestic animals, should raise concerns about the potential risk of inter-species transmission events leading to the possibility of more pathogenic viruses crossing into human populations.

A recent study of microRNA expression in porcine parvovirus infection describes upregulation of microRNAs that are known to be immune system regulators ^52^. In the longer term, this kind of study may provide further insights into the nature and mechanism of host-virus interactions.

## Discussion and conclusions

As this review illustrates, our understanding of PARV4 continues to advance gradually, but there are still many important unresolved questions, and the significance of this infection to human health remains uncertain. The striking difference in population epidemiology in developed and developing countries currently remains unexplained, and it is uncertain to what extent differences in virus properties or host characteristics account for the marked differences in population seroprevalence. For the former, it is plausible that genotype-specific amino acid substitutions underpin differences in the route, mechanism, or efficiency of transmission. From the host side, it is possible that differences in human genetics, prevalent co-infections, or environmental, behavioural, or iatrogenic exposure account for differences in seroprevalence and risk factors between continents.

Ongoing diligent scrutiny of carefully characterised clinical cohorts will be required to cement any associations with human disease; this may require larger collaborative efforts than have been undertaken to date. An *in vitro* system is still needed to help us understand the biology of PARV4 in more detail, including characterisation of tissue tropism, productivity of cellular infection, the influence of co-infecting viruses, and the mechanisms underlying viral suppression by the immune system.

This review highlights a number of compelling arguments in favour of advancing our understanding of PARV4. Firstly, this agent is endemic in some human populations; even if pathology emerges in only a small subset of infected individuals, this may still be important in terms of overall case numbers. Putative associations with significant pathology may be particularly relevant for certain vulnerable groups, including those with other chronic viral infection (HIV, HBV, or HCV), those with solid organ or bone marrow transplantation, and recipients of human blood or tissue transplant ^11, 35^. Secondly, we know that viral infections are relevant to the host in more diverse ways than just the effects of primary infection; they may shape immune ontogeny, influence the outcomes of infection with other pathogens ^54^, and drive or control oncogenesis. Understanding viral biology also allows us to draw on these agents as a resource for vaccine design, as exemplified by the adenovirus constructs used in vaccine trials for HIV ^55, 56^ and HCV ^57^.

Therefore, whether PARV4 turns out to be an innocent bystander or an elusive human pathogen, there are robust reasons for advocating ongoing clinical and research efforts to further our understanding of epidemiology and the current and future potential significance to human health.

## Abbreviations

B19V, parvovirus B19; BBV, blood-borne virus; CSF, cerebrospinal fluid; HBV, hepatitis B virus; HCV, hepatitis C virus; HIV, human immunodeficiency virus; HTLV, human T-cell lymphotrophic virus; NS, non-structural; PARV4, human parvovirus 4; PCR, polymerase chain reaction; PWID, person who injects drugs.

## Supplementary data

**(A) PARV4 ORF-1 amino acid sequence; (B) PARV4 ORF-2 amino acid sequence.**

## References

[1] Jones MS Kapoor A Lukashov VV: New DNA viruses identified in patients with acute viral infection syndrome. *J Virol.* 2005;79(13):8230–6. 10.1128/JVI.79.13.8230-8236.2005 15956568 PMC1143717

[2] Matthews PC Malik A Simmons R: PARV4: an emerging tetraparvovirus. *PLoS Pathog.* 2014;10(5):e1004036. 10.1371/journal.ppat.1004036 24789326 PMC4006886

[3] Lau SK Woo PC Tse H: Identification of novel porcine and bovine parvoviruses closely related to human parvovirus 4. *J Gen Virol.* 2008;89(Pt 8):1840–8. 10.1099/vir.0.2008/000380-0 18632954

[4] Sharp CP Vermeulen M Nébié Y: Changing epidemiology of human parvovirus 4 infection in sub-Saharan Africa. *Emerg Infect Dis.* 2010;16(10):1605–7. 10.3201/eid1610.101001 20875290 PMC3294412

[5] Simmonds P Manning A Kenneil R: Parenteral transmission of the novel human parvovirus PARV4. *Emerg Infect Dis.* 2007;13(9):1386–8. 10.3201/eid1309.070428 18252117 PMC2857296

[6] Fryer JF Lucas SB Padley D: Parvoviruses PARV4/5 in hepatitis C virus-infected patient. *Emerg Infect Dis.* 2007;13(1):175–6. 10.3201/eid1301.061358 17370542 PMC2725819

[7] Simmons R Sharp C Levine J: Evolution of CD8+ T cell responses after acute PARV4 infection. *J Virol.* 2013;87(6):3087–96. 10.1128/JVI.02793-12 23283958 PMC3592128

[8] Simmons R Sharp C McClure CP: Parvovirus 4 infection and clinical outcome in high-risk populations. *J Infect Dis.* 2012;205(12):1816–20. 10.1093/infdis/jis291 22492853 PMC3357136

[9] Lurcharchaiwong W Chieochansin T Payungporn S: *Parvovirus* 4 (PARV4) in serum of intravenous drug users and blood donors. *Infection.* 2008;36(5):488–91. 10.1007/s15010-008-7336-4 18759058

[10] Maple PA Beard S Parry RP: Testing UK blood donors for exposure to human parvovirus 4 using a time-resolved fluorescence immunoassay to screen sera and Western blot to confirm reactive samples. *Transfusion.* 2013;53(10 pt 2):2575–84. 10.1111/trf.12278 23721256

[11] Sharp CP Lail A Donfield S: Virologic and clinical features of primary infection with human parvovirus 4 in subjects with hemophilia: frequent transmission by virally inactivated clotting factor concentrates. *Transfusion.* 2012;52(7):1482–9. 10.1111/j.1537-2995.2011.03420.x 22043925

[12] Servant-Delmas A Laperche S Lionnet F: Human parvovirus 4 infection in low- and high-risk French individuals. *Transfusion.* 2014;54(3):744–5. 10.1111/trf.12512 24617588

[13] Simmonds P Douglas J Bestetti G: A third genotype of the human parvovirus PARV4 in sub-Saharan Africa. *J Gen Virol.* 2008;89(pt 9):2299–302. 10.1099/vir.0.2008/001180-0 18753240

[14] Lavoie M Sharp CP Pépin J: Human parvovirus 4 infection, Cameroon. *Emerg Infect Dis.* 2012;18(4):680–3. 10.3201/eid1804.110628 22469425 PMC3309673

[15] Drexler JF Reber U Muth D: Human parvovirus 4 in nasal and fecal specimens from children, Ghana. *Emerg Infect Dis.* 2012;18(10):1650–3. 10.3201/eid1810.111373 23018024 PMC3471610

[16] Panning M Kobbe R Vollbach S: Novel human parvovirus 4 genotype 3 in infants, Ghana. *Emerging Infect Dis.* 2010;16(7):1143–6. 10.3201/eid1607.100025 20587191 PMC3321913

[17] Rosenfeldt V Norja P Lindberg E: Low Prevalence of Parvovirus 4 in HIV-infected Children in Denmark. *Pediatr Infect Dis J.* 2015;34(7):761–2. 10.1097/INF.0000000000000642 25545184

[18] von Linstow ML Rosenfeldt V Lindberg E: Absence of novel human parvovirus (PARV4) in Danish mothers and children. *J Clin Virol.* 2015;65:23–5. 10.1016/j.jcv.2015.01.021 25766982

[19] Asiyabi S Nejati A Shoja Z: First report of human parvovirus 4 detection in Iran. *J Med Virol.* 2016;88(8):1314–8. 10.1002/jmv.24485 26812938

[20] Slavov SN Kashima S Rocha-Junior MC: Human parvovirus 4 in Brazilian patients with haemophilia, beta-thalassaemia major and volunteer blood donors. *Haemophilia.* 2015;21(1):e86–8. 10.1111/hae.12564 25311656

[21] Slavov SN Otaguiri KK Smid J: Human parvovirus 4 prevalence among HTLV-1/2 infected individuals in Brazil. *J Med Virol.* 2017;89(4):748–752. 10.1002/jmv.24673 27589576

[22] Tolfvenstam T Norbeck O Ohrmalm L: No evidence of presence of parvovirus 4 in a Swedish cohort of severely immunocompromised children and adults. *PLoS One.* 2012;7(9):e46430. 10.1371/journal.pone.0046430 23050026 PMC3458858

[23] Matthews PC Sharp CP Malik A: Human parvovirus 4 infection among mothers and children in South Africa. *Emerging Infect Dis.* 2015;21(4):713–5. 10.3201/eid2104.141545 25812109 PMC4378500

[24] Chen MY Yang SJ Hung CC: Placental transmission of human parvovirus 4 in newborns with hydrops, Taiwan. *Emerging Infect Dis.* 2011;17(10):1954–6. 10.3201/eid1710.101841 22000381 PMC3310659

[25] Fryer JF Delwart E Hecht FM: Frequent detection of the parvoviruses, PARV4 and PARV5, in plasma from blood donors and symptomatic individuals. *Transfusion.* 2007;47(6):1054–61. 10.1111/j.1537-2995.2007.01235.x 17524097

[26] Manning A Willey SJ Bell JE: Comparison of tissue distribution, persistence, and molecular epidemiology of parvovirus B19 and novel human parvoviruses PARV4 and human bocavirus. *J Infect Dis.* 2007;195(9):1345–52. 10.1086/513280 17397006 PMC7109978

[27] Schneider B Fryer JF Reber U: Persistence of novel human parvovirus PARV4 in liver tissue of adults. *J Med Virol.* 2008;80(2):345–51. 10.1002/jmv.21069 18098166

[28] Schneider B Fryer JF Oldenburg J: Frequency of contamination of coagulation factor concentrates with novel human parvovirus PARV4. *Haemophilia.* 2008;14(5):978–86. 10.1111/j.1365-2516.2008.01800.x 18565125

[29] Fryer JF Hubbard AR Baylis SA: Human parvovirus PARV4 in clotting factor VIII concentrates. *Vox Sang.* 2007;93(4):341–7. 10.1111/j.1423-0410.2007.00979.x 18070279

[30] Norja P Hokynar K Aaltonen LM: Bioportfolio: lifelong persistence of variant and prototypic erythrovirus DNA genomes in human tissue. *Proc Natl Acad Sci U S A.* 2006;103(19):7450–3. 10.1073/pnas.0602259103 16651522 PMC1464359

[31] Yang SJ Hung CC Chang SY: Immunoglobulin G and M antibodies to human parvovirus 4 (PARV4) are frequently detected in patients with HIV-1 infection. *J Clin Virol.* 2011;51(1):64–7. 10.1016/j.jcv.2011.01.017 21353629

[32] Benjamin LA Lewthwaite P Vasanthapuram R: Human parvovirus 4 as potential cause of encephalitis in children, India. *Emerg Infect Dis.* 2011;17(8):1484–7. 10.3201/eid1708.110165 21801629 PMC3381555

[33] Chen MY Hung CC Lee KL: Detection of human parvovirus 4 viremia in the follow-up blood samples from seropositive individuals suggests the existence of persistent viral replication or reactivation of latent viral infection. *Virol J.* 2015;12:94. 10.1186/s12985-015-0326-0 26088443 PMC4480887

[34] Simmons R Sharp C Sims S: High frequency, sustained T cell responses to PARV4 suggest viral persistence *in vivo*. *J Infect Dis.* 2011;203(10):1378–87. 10.1093/infdis/jir036 21502079 PMC3080894

[35] Vallerini D Barozzi P Quadrelli C: Parvoviruses in blood donors and transplant patients, Italy. *Emerg Infect Dis.* 2008;14(1):185–6. 10.3201/eid1401.070610 18258108 PMC2600167

[36] Corcioli F Zakrzewska K Fanci R: Human parvovirus PARV4 DNA in tissues from adult individuals: a comparison with human parvovirus B19 (B19V). *Virol J.* 2010;7:272. 10.1186/1743-422X-7-272 20950445 PMC2965155

[37] Vuorinen T Lammintausta K Kotilainen P: Presence of parvovirus B19 DNA in chronic urticaric and healthy human skin. *J Clin Virol.* 2002;25(2):217–21. 10.1016/S1386-6532(02)00012-4 12367657

[38] Tu M Liu F Chen S: Role of capsid proteins in parvoviruses infection. *Virol J.* 2015;12:114. 10.1186/s12985-015-0344-y 26239432 PMC4524367

[39] Prakash S Jain A Seth A: Complete genome sequences of two isolates of human parvovirus 4 from patients with acute encephalitis syndrome. *Genome Announc.* 2015;3(1): pii: e01472-14. 10.1128/genomeA.01472-14 25635010 PMC4319504

[40] Kafatos G Andrews NJ McConway KJ: Is it appropriate to use fixed assay cut-offs for estimating seroprevalence? *Epidemiol Infect.* 2016;144(4):887–95. 10.1017/S0950268815001958 26311119

[41] Vaisanen E Lahtinen A Eis-Hubinger AM: A two-step real-time PCR assay for quantitation and genotyping of human parvovirus 4. *J Virol Methods.* 2014;195:106–11. 10.1016/j.jviromet.2013.10.011 24134943

[42] Sharp CP Lail A Donfield S: High frequencies of exposure to the novel human parvovirus PARV4 in hemophiliacs and injection drug users, as detected by a serological assay for PARV4 antibodies. *J Infect Dis.* 2009;200(7):1119–25. 10.1086/605646 19691429 PMC2914696

[43] Baylis SA Tuke PW Miyagawa E: Studies on the inactivation of human parvovirus 4. *Transfusion.* 2013;53(10 Pt 2):2585–92. 10.1111/trf.12372 24032592

[44] Norja P Lassila R Makris M: Parvovirus transmission by blood products - a cause for concern? *Br J Haematol.* 2012;159(4):385–93. 10.1111/bjh.12060 23025427

[45] Delwart E: Human parvovirus 4 in the blood supply and transmission by pooled plasma-derived clotting factors: does it matter? *Transfusion.* 2012;52(7):1398–403. 10.1111/j.1537-2995.2012.03721.x 22780892 PMC3666916

[46] Fryer JF Kapoor A Minor PD: Novel parvovirus and related variant in human plasma. *Emerg Infect Dis.* 2006;12(1):151–4. 10.3201/eid1201.050916 16494735 PMC3291395

[47] Touinssi M Reynaud-Gaubert M Gomez C: Parvovirus 4 in French in-patients: a study of hemodialysis and lung transplant cohorts. *J Med Virol.* 2011;83(4):717–20. 10.1002/jmv.22003 21328388

[48] Miranda C Coelho C Vieira-Pinto M: Porcine hokovirus in wild boar in Portugal. *Arch Virol.* 2016;161(4):981–4. 10.1007/s00705-015-2730-6 26711454

[49] Xu F Pan Y Wang M: First detection of ungulate tetraparvovirus 1 (bovine hokovirus 1) in domestic yaks in northwestern China. *Arch Virol.* 2016;161(1):177–80. 10.1007/s00705-015-2638-1 26483281

[50] Souza CK Streck AF Goncalves KR: Phylogenetic characterization of the first Ungulate tetraparvovirus 2 detected in pigs in Brazil. *Braz J Microbiol.* 2016;47(2):513–7. 10.1016/j.bjm.2016.01.025 26991274 PMC4874618

[51] Ni J Qiao C Han X: Identification and genomic characterization of a novel porcine parvovirus (PPV6) in China. *Virol J.* 2014;11:203. 10.1186/s12985-014-0203-2 25442288 PMC4265361

[52] Li X Zhu L Liu X: Differential expression of micrornas in porcine parvovirus infected porcine cell line. *Virol J.* 2015;12:128. 10.1186/s12985-015-0359-4 26290078 PMC4545981

[53] Heeney JL: Zoonotic viral diseases and the frontier of early diagnosis, control and prevention. *J Intern Med.* 2006;260(5):399–408. 10.1111/j.1365-2796.2006.01711.x 17040245

[54] Adland E Klenerman P Goulder P: Ongoing burden of disease and mortality from HIV/CMV coinfection in Africa in the antiretroviral therapy era. *Front Microbiol.* 2015;6:1016. 10.3389/fmicb.2015.01016 26441939 PMC4585099

[55] Gray GE Allen M Moodie Z: Safety and efficacy of the HVTN 503/Phambili study of a clade-B-based HIV-1 vaccine in South Africa: a double-blind, randomised, placebo-controlled test-of-concept phase 2b study. *Lancet Infect Dis.* 2011;11(7):507–15. 10.1016/S1473-3099(11)70098-6 21570355 PMC3417349

[56] Fitzgerald DW Janes H Robertson M: An Ad5-vectored HIV-1 vaccine elicits cell-mediated immunity but does not affect disease progression in HIV-1-infected male subjects: results from a randomized placebo-controlled trial (the Step study). *J Infect Dis.* 2011;203(6):765–72. 10.1093/infdis/jiq114 21343146 PMC3119328

[57] Barnes E Folgori A Capone S: Novel adenovirus-based vaccines induce broad and sustained T cell responses to HCV in man. *Sci Transl Med.* 2012;4(115):115ra1. 10.1126/scitranslmed.3003155 22218690 PMC3627207

[58] Taylor S Lopez P Weckx L: Respiratory viruses and influenza-like illness: Epidemiology and outcomes in children aged 6 months to 10 years in a multi-country population sample. *J Infect.* 2017;74(1):29–41. 10.1016/j.jinf.2016.09.003 27667752 PMC7112512

[59] Kailasan S Agbandje-McKenna M Parrish CR: Parvovirus Family Conundrum: What Makes a Killer? *Annu Rev Virol.* 2015;2(1):425–50. 10.1146/annurev-virology-100114-055150 26958923

