# Supplementary material for: Human parvovirus 4 ‘PARV4’ remains elusive despite a decade of study
Source: F1000Res. 2017 Jan 27;6:82. [Version 1] doi: 10.12688/f1000research.9828.1 (PMC5288687; doi:10.12688/f1000research.9828.1)
Supplement: Supplementary file 1 [file f1000research-6-10596-s0000.tgz › d14f5213-8638-4bf4-8d13-6cfb1752c664.pdf]

Supplementary Data 1: (A) PARV4 ORF-1 amino acid sequence; (B) PARV4 ORF-2 amino acid sequence

(A) Human Parvovirus 4 Open Reading Frame 1: Consensus and Variant sequences

Sequences downloaded from NCBI September 2016, specifying length 600-700 amino acids; n=17.
Aligned using Cobalt on NCBI;
Consensus sequence created on 'consensus creator' at Los Alamos HIV databases;
Numbering is provided for amino acid position relative to consensus.

```
CONSENSUS   1     MDAPAWIAVL QIPTGFLSNP ANWRDWDGLQ RPRNLLADDW PIQELRESVP   50
VARIANT           .......... .......... .........H .......... ..........

CONSENSUS   51    LFDHAVNLGY CILQQLFASH AVTLPCRVKP SMFLQLEPSS GEENEMHYHL   100
VARIANT           F......... .T........ .......... ..S....... ..D.......

CONSENSUS   101   VVNQADMVGR ECSNWLRTWk VFMAGYLVAP VWTLSWNIRK TRQGRLYQAD   150
VARIANT           .I........ ....C..... .......... T......... ..........

CONSENSUS   151   MSFVKNYLLP KLPLNDCYYA WTNIDRFEAA VLSVRNRQLS GPQGAIALPF   200
VARIANT           .......... .......... .......... .......... ..........

CONSENSUS   201   TDAPPQAPAA EGVPPTMAGK GTQRFMDLID WLVENGIATE KRWLSVNKLS   250
VARIANT           S..Q.RT... ..I....... .......... .......... ..........

CONSENSUS   251   YRSFLGSSGG VLQAKNALQI AKREMVLAHP LLGYLTKNAS TFEESNKVAQ   300
VARIANT           .......... .......... .......... .......... AY..G.....

CONSENSUS   301   LFSLNGYNPV DAAWYFAAWA RGVWPKRRAI WLWGPASTGK TLLAAAIANL   350
VARIANT           .......... .......... .......... .......... ..........

CONSENSUS   351   SPSYGCVNWT NQNFPFNDCH CQSLVWWEEG RMTENIVEVA KAVLGGAPVR   400
VARIANT           .......... .......... .......... .......... ..........

CONSENSUS   401   LDVKNKGSED YIPTCVIITS NGDLTVTVDG PVVSTQHQEA LQTRITMFQF   450
VARIANT           .......... .......... .......... .........T ..........

CONSENSUS   451   QRMVPDGLAP LPEEEVRSFF KLGEQELNMK GTPPEEFRVP RNFDKQPMAS   500
VARIANT           .......... .......... .......... .......K.. ..........

CONSENSUS   501   TSNLPKALCA PIDENQVQWD SEDDWFPPPT QKKRREVQET PPTTPSEVIE   550
VARIANT           .....R.... .MED.P.... .......... .....AL... ..R....I..

CONSENSUS   551   LSSPSPLAdA PPRTPDSLGE LSLTPTSVSQ IVSAPFPEET AERYGAGDIE   600
VARIANT           ......V... ..T....... .......I.. ........A. ..........

CONSENSUS   601   SFWSEHVFDA DWATRLHICP PGGPRPYGLF WTYLWSREFW RFKQSVSRSE   650
VARIANT           A......... .......... ....K..... .......... .....L....

CONSENSUS   651   AHLINRRFIW AWW      663
VARIANT           ...V...... ...
```

(B) Human Parvovirus 4 Open Reading Frame 2: Consensus and Variant sequences

Sequences downloaded from NCBI September 2016, specifying length 800-1000 amino acids; n=16.
Aligned using Cobalt on NCBI;
Consensus sequence created on 'consensus creator' at Los Alamos HIV databases;
Numbering is provided for amino acid position relative to consensus.

```
CONSENSUS     1     MSAADAYRPG GKLPLDELMQ RMNRAIPVGP EPSSQANRGG GPYQTHFAIG   50
VARIANTS            .......... .......... .......... ....P..... .T........

CONSENSUS     51    IMYSKAFQGL LRFAnALPAE LSPVKQLVNQ LENYRRKTSD TRVWYRVYLD   100
VARIANTS            .......... .K......P. .N.....I.. ..H....... .........N

CONSENSUS     101   MTRLLISVAP PGAANKLRQA AAGITHSKAP NAESLRGIVR FAAAAFVPTV   150
VARIANTS            .......... .......... ....S..... .......... ..........

CONSENSUS     151   ENIDRFFEDS LTNFAKEDLd TWQQLHEQFI KLFHPPDVGV HLVSDSRDEG   200
VARIANTS            .......... .......... ..K....... .......... ..I.......

CONSENSUS     201   ADSLVEPDLE RPAGGGLTLP GYNYVGPGNP LDSGPPQGPV DEAAKHHDER   250
VARIANTS            ...I...... .......... .......... ........S. ..T....N..

CONSENSUS     251   YAEMIEHGDI PYLHGHGADR LMNKELEEKE RRGDITHLAD VVVGNAIRGL   300
VARIANTS            .....K.... ..F....... .......... .....S..T. ..........

CONSENSUS     301   WQAKETVGDI ADVQLSQVLP PAPPSSDQQP AYSAGEPSAK KARIGTPEES   350
VARIANTS            .......... .......... .......... .......LP. .......DK.

CONSENSUS     351   EPALLLQSHT NTMSVEPAGG GGGVKVKAQW IGGTSFSDSV VITSHTRTSM   400
VARIANTS            D.T....PN. D......... ...L...S.. .......... ..........
VARIANTS            ........D. H......... .......... .......... ..........

CONSENSUS     401   LADRGGYVPV YKQGSHVDSS QPVMGMKTPY SYIDVNALSA HFTPRDFQQL   450
VARIANTS            .......... ......I... .......... .......... ..........

CONSENSUS     451   LDEYDEIKPK SLTIAISAIV IKDVATNQTG TTVSDSASGG ITVFADDSYD   500
VARIANTS            .......... .......... .......... .N........ ..I.......

CONSENSUS     501   YPYVLGHNQD TLPGHLPGEN YVLPQYGYIT RGREIDQQNS IVAISDHKTE   550
VARIANTS            .......... .......... .......... .......... ..........

CONSENSUS     551   LFFLEHHDAE CLGTGDHWSH HYEFPDDLPW RKLSTPNQTL YARHNPIPSS   600
VARIANTS            .......... .........R .......... .......... ..........

CONSENSUS     601   RLAIMTGVDN DGTAIWKRPE GMVGRLPLN YVPGPALMMP TDTQIRNTTF   650
VARIANTS            .......... ..A....... .L........ .......... ..........

CONSENSUS     651   RDPVAIGNPA TSDRYSVAPL VHQPWSVRTE EWLANKTDYA VHNYLGGVAY   700
VARIANTS            .......... .......... .......... .........S ..........
```

```
CONSENSUS       701   TRRKHEESYD KHEEDRDGRV TNPSRVVQID GDLAAPHVGH TFFVPGHTRV   750
VARIANTS              .......... .......... .......... .......... ..........

CONSENSUS       751   TSGGTDTVYS PKLYQEPVFP LFPGAVWNPN PLSYDCQIWT KIPNTECHFF   800
VARIANTS              ........H. .......... .......... .......... ..........

CONSENSUS       801   AQYPLLGGWG VLTPPPMIFV KLRSQPGPPS PGAHTVPQSN LNQYAIFHLH   850
VARIANTS              .......... .F........ .......... .......... ..........

CONSENSUS       851   YSMQFLVKRR KRSRRHNPEK PAPFPTTDSG RMPFTLANSL KDPNTPVYEV   900
VARIANTS              .......... ...H...... .......... .......... ........G.

CONSENSUS       901   PSDQWIARNY SHLL    914
VARIANTS              .......... ....
```
